# Effects of golf instructors’ professional certification levels on amateur golfers’ perception of instructor expertise, instructor credibility, and lesson participation intention: testing placebo and nocebo effects

**DOI:** 10.3389/fpsyg.2024.1361470

**Published:** 2024-03-12

**Authors:** Hye Jin Yang, Ji-Hye Yang, Si Cheol Jung, Chulhwan Choi, Chul-Ho Bum

**Affiliations:** ^1^Department of Physical Education, Graduate School, Kyung Hee University, Yongin-si, Gyeonggi-do, Republic of Korea; ^2^Department of Physical Education, Gachon University, Seongnam-si, Gyeonggi-do, Republic of Korea; ^3^College of Physical Education, Kyung Hee University, Yongin-si, Gyeonggi-do, Republic of Korea

**Keywords:** golf, instructor expertise, instructor credibility, lesson participation intention, placebo, nocebo

## Abstract

This study investigated the differences in amateur golfers’ perceptions of instructor expertise, instructor credibility, and lesson participation intention depending on the golf instructor’s certification level to investigate whether placebo and nocebo effects exist depending on the certification level. Accordingly, the study analyzed 153 amateur golfers with at least 1 year of playing experience, and the results were as follows: First, there was a difference in the perception of instructor expertise among amateur golfers depending on the golf instructor’s certification level. Specifically, there were significant differences in perceived performance and teaching skills but no differences in personality and emphasis on basic principles. Second, the participants reported significant differences in their perceptions of instructor credibility depending on the instructor’s certification level. Instructor credibility of the tournament professional group was the highest, whereas that of the amateur group was the lowest. Third, the results showed differences in lesson participation intention among amateur golfers depending on the instructor’s certification level. Lesson participation intention was higher for semi-professional and tournament professional instructors than for amateur instructors. These results verified the presence of psychological biases, such as placebo/nocebo effects, that result in differences in the perception of instructor expertise, instructor credibility, and lesson participation intention depending on the certification level of instructors. Additionally, based on the data obtained from this study, further research is required to improve the performance of golf instructors and create an efficient teaching environment.

## 1 Introduction

The golfing population in South Korea reached 4.69 million people in 2017, which is an increase of 860,000 from the previous year, and it has recorded a remarkable average growth rate of 11.6% for six consecutive years (Kim, 2018). Owing to the increase in the number of new golfers, the number of enthusiasts willing to take golf lessons is also increasing. Notably, among amateur golfers, nine out of ten have received golf lessons owing to the perception that “golf is difficult to master and basic posture is essential” (Shin, 2017).

Two significant implications can be drawn from this information. First, owing to the nature of golf, which is a sport that is difficult to master compared to other leisure sports, the instructor’s role is critical. Kelly et al. (2010) stated that a good golf swing, which is essential in lowering the score, is made up of various aspects, such as flexibility and stability, thereby making it challenging to master within a short period. Additionally, amateur golfers recognize the challenge of correcting one’s posture once a golf swing is off, which emphasizes the importance of receiving regular lessons from an instructor (Jang, 2019). Therefore, both well-known elite athletes and the general population who enjoy sports require instructors.

Efficient coaching in all sports fields, including golf, is recognized as a crucial issue in both the relevant academic and practical fields involving the coaching of athletes or students (Gould et al., 2002; Thelwell et al., 2008). Among the many factors required from a sports instructor, the most important is “expertise,” which is a concept that is viewed differently across different fields and among scholars. Swanson (2001) defined expertise as having the best capabilities necessary to perform one’s job in one’s field. Hoffman et al. (1995) described the necessary components necessary to possess expertise as rich knowledge, competent skills, and the attitude necessary to perform a task. Moreover, field experience and problem-solving ability (Herling, 1998; Swanson and Holton, 2001), flexibility and an achievement-oriented attitude (Heijden, 2000), self-confidence or intuition, and efforts for self-improvement (Germain, 2006) are also considered components of expertise.

Instructor expertise in the field of sports can be analyzed from an integrated perspective that includes knowledge, game operation methods, tactics and strategy development, as well as specific coaching contexts for achieving the ultimate goal of performance improvement (Riemer and Chelladurai, 1995; Côté and Gilbert, 2009). Furthermore, encouraging active participation through motivation (Grant, 2006; Liljenstrand and Nebeker, 2008) and efforts for building mutual relationships (Gyllensten and Palmer, 2007) have also been mentioned. These characteristics of coaches with expertise have a significant impact on athletes’ trust in their instructors (Zhang and Chelladurai, 2013).

Trust refers to one’s faith and confidence in another being and the sense of physical and mental stability one experiences when they are with another person (Bede et al., 2021). Trust has been described as the degree to which one believes and fully accepts the words, actions, and behaviors of others (McAllister, 1995; Mcknight et al., 1998). Although it is difficult to find a standardized definition of trust, many people agree on its importance (Hosmer, 1995; Dirks, 2000). Ultimately, trust results in positive outcomes (Smyth et al., 2010) by fostering understanding and seamless interaction between two parties (Bstieler, 2006).

The positive effects of trust have been demonstrated in sports. According to Zhang and Chelladurai (2013), trusting the instructor is a precursor to being immersed in the instructions, which further helps to enhance performance. Conversely, even if an instructor possesses excellent coaching behaviors or strategies, if the athlete does not trust the instructor, the effectiveness of coaching is likely to be limited (Dirks, 2000; Furrer and Skinner, 2003).

This study selected people who genuinely enjoy golf as the study participants for two reasons. First, most research related to coaching behavior focuses on elite athletes, and research on coaching behavior related to amateurs is lacking, despite its importance. Second, golf was chosen based on the aforementioned characteristic of the sport as having a higher lesson rate than other sports. From a new perspective, this study verified the logical causality of the basic premise that “a great player makes a great coach” and investigated whether evaluating coaches based on this belief is a logical error.

Challenging the premise that “a great player makes a great coach,” this study verified the psychological errors (placebo and nocebo effects) involved in judging one’s ability based on one’s background as an instructor. The findings of this study are meaningful in that they provide data for developing the practical abilities required among instructors and creating an efficient coaching environment as well as a satisfying lesson environment for golfers.

## 2 Methods

### 2.1 Participants

In this study, people from the Republic of Korea aged 20 years or older and with more than a year of golf experience or golf lessons experience were selected as the study population, and non-probability sampling and purpose sampling were applied. Those who had never played golf, had never taken lessons, or had not played golf for less than a year were excluded from this study. The study involved only the people who provided consent to participate. Online and offline data collection was conducted at an outdoor golf practice range, a university practice range, and a 4-year university located in the Republic of Korea through purposeful sampling. Detailed information is reported in Table 1. The Institutional Review Board (IRB) of Kyung Hee University provided ethical approval in accordance with the Declaration of Helsinki.

**Table 1:** Descriptive statistics by groups.

		Group 1 Amateur	Group 2 Semi-professional	Group 3 Professional
Sex	Male	37 (72.5%)	34 (66.7%)	41 (80.4%)
	Female	14 (27.5%)	17 (33.3%)	10 (19.6%)
Age	20 s	12 (23.5%)	6 (11.8%)	1 (2.0%)
	30 s	12 (19.6%)	17 (33.3%)	9 (17.6%)
	40 s	14 (27.5%)	12 (23.5%)	25 (49.0%)
	Over 50 s	15 (29.4%)	16 (31.4%)	19 (31.4%)
Golf lesson experience	Yes	37 (72.5%)	49 (96.1%)	44 (86.3%)
	No	14 (27.5%)	2 (3.9%)	7 (13.7%)
Golf experience	Less than 1 year	15 (29.4%)	7 (13.7%)	12 (23.5%)
	1–5 years	12 (23.5%)	31 (60.8%)	15 (29.4%)
	5–10 years	12 (23.5%)	6 (11.8%)	15 (29.4%)
	10–20 years	9 (17.6%)	5 (9.8%)	7 (13.7%)
	More than 20 years	3 (5.9%)	2 (3.9%)	2 (3.9%)
Golf handicap	Less than 10	4 (7.8%)	1 (2.0%)	5 (9.8%)
	10–15	13 (25.5%)	10 (19.6%)	11 (21.6%)
	15–20	14 (27.5%)	14 (27.5%)	19 (37.3%)
	More than 20	11 (21.6%)	14 (27.5%)	13 (25.5%)
	I do not know	9 (17.6%)	12 (23.5%)	3 (5.9%)
Frequency of golf practice	Less than once per month	2 (3.9%)	4 (7.8%)	5 (9.8%)
	Once or twice per month	16 (31.4%)	11 (21.6%)	14 (27.5%)
	Once per week	13 (25.5%)	10 (19.6%)	15 (29.5%)
	More than twice per week	12 (23.5%)	20 (39.2%)	15 (29.5%)
	Almost everyday	8 (15.7%)	6 (11.8%)	2 (3.9%)
Total		51 (100%)	51 (100%)	51 (100%)

### 2.2 Study design and procedure

Before the filming of the lesson video, this study conducted a discussion involving the common issues amateur golfers may encounter regarding swing posture, which was handled by four professionals inform the Korean Professional Golf Association with over 3 years of teaching experience. Based on the issues regarding swing posture, the lesson content was structured in detail to ensure that the participants could determine the factors that would indicate instructor expertise. An instructor selected by the researchers filmed a 10-min lesson video (Table 2) featuring the amateur golfers’ problems and the corresponding methods for improvement. During filming, the instructor followed the script without expressing their personal opinions or coaching methods. The lesson content was the same for all three groups of amateur golfers. Subsequently, the lesson video was sent to the amateur golfers who expressed interest in participating in the study, both online and offline (through golf practice facilities in C and S cities).

**Table 2:** Golf lesson video contents.

Lesson script	Details
1. Opening	Instructor’s self-introduction and purpose of the lesson
2. Problem: Early extension Maintaining spine and hip angles	Explanation and cause of early extension Compensations caused by early extension Importance of maintaining spine and hip angles
3. Instructor’s demonstration	Front/side swing
4. Correction of early extension	Swing with hips against the wall Swing while pressing a gym ball with hips
5. Problem: Incorrect rotation during back swing Back swing coiling	Explanation of back swing coiling Compensations and problems caused by incorrect rotation Effects of back swing coiling
6. Instructor’s demonstration	Front/side swing
7. Correct rotation and correction method for back swing	Rotate with the club on the right shoulder and extend arms after rotation
8. Closing	Summary of the lesson and importance of practice

To determine the sample size for this study, the analysis used G*Power 3.1.9.7. A medium effect size (0.07) and acceptable power (0.95) were set for the program. A total sample size of 153 participants was calculated after entering the number of groups (3) representing the participant groups, which were divided into two groups by one independent variable, and the response variables (3) representing the dependent variables (instructor expertise, instructor credibility, and lesson participation intention). The amateur golfers in the three groups (51 participants in each group) received the lesson video and were told that the coach was a tournament professional (tour pro) (Group 1), a semi-professional (semi-pro) (Group 2), and an aspiring pro (Group 3). After watching the lesson video, the participants completed a questionnaire regarding instructor expertise, instructor credibility, and lesson participation intention.

### 2.3 Instruments

The dependent variables used to compare and analyze the psychological errors of the golf participants were instructor expertise, instructor credibility, and lesson participation intention. For instructor expertise, this study modified the factors previously used by Kim D. H. (2013), Hwang (2019), and Lee (2009) to analyze participants’ perceptions of instructor expertise. This study developed 14 survey questions based on four sub-factors (performance, teaching skills, personality and ethics, insight, and analytical ability). For instructor credibility, this study composed four survey questions by applying the factors used by Kim J. S. (2013) to analyze the causal relationships between controlling coaching behavior, group cohesion, instructor credibility, and exercise satisfaction as a single factor. Finally, for lesson participation intention, this study restructured the single factor revised by Jung (2008) based on the measurement methods of behavioral intention reported by McAuley (1993) and Wilson and Rodgers (2001) into five survey questions to fit this study. All the items were rated using a Likert scale ranging from 1 = *not at all* to 5 = *extremely*.

### 2.4 Data analysis

Based on SPSS/AMOS 23.0 for the data collected, this study conducted an exploratory factor analysis using a statistical criterion of eigenvalue 1.00 or greater to identify the structure of the factors used in the study. This study (a) derived basic socio-demographic results, including gender and age, using descriptive statistics and (b) used Cronbach’s alpha coefficients to verify the reliability of the data, with a statistical criterion of >0.7, as suggested by Nunnally and Bernstein (1994). After analyzing the collected data, this study conducted the main data analysis to address the research problem. A one-way multivariate analysis of variance (MANOVA) was performed to compare and analyze the differences in instructor expertise, instructor credibility, and lesson participation intention among the three groups (tour pro, semi-pro, and aspiring pro).

## 3 Results

### 3.1 Validity and reliability

Exploratory factor analysis (EFA) was implemented before the statistical analyses to verify the validity of the scale applied in this study. Specifically, sub-factors with an eigenvalue of 1.00 or greater were selected based on a Varimax rotation to determine the factor structure of the dependent variable (instructor expertise). Two factors (instructor credibility and lesson participation intention) were excluded from the EFA as single-scale factors. The detailed results of the EFA are listed in Table 3.

**Table 3:** Results of EFA.

Factors	Survey items	1	2	3	4
Performance	This instructor has a great swing.	**0.871**	0.322	−0.025	0.115
	This instructor has a variety of swing techniques.	**0.870**	0.287	0.100	0.078
	This instructor is an outstanding athlete.	**0.859**	0.268	0.114	0.103
	This instructor does a great job of demonstrating the swing.	**0.842**	0.315	0.131	0.149
Teaching skills	This instructor effectively utilizes precise instruction.	0.264	**0.876**	0.155	0.110
	This instructor provides easy-to-follow instructions.	0.346	**0.821**	0.118	0.142
	This instructor encourages and motivates golfers.	0.358	**0.813**	0.174	0.146
	This instructor offers solutions for golfers.	0.356	**0.760**	0.218	0.134
Personality and ethics	This instructor is professional.	0.122	0.029	**0.850**	0.215
	This instructor is passionate about teaching.	−0.006	0.241	**0.839**	0.178
	This instructor is responsible.	0.132	0.232	**0.810**	0.245
Basic principle	This instructor emphasizes basic principle for the swing.	0.104	0.082	0.100	**0.869**
	This instructor emphasizes the principles of the swing.	0.059	0.177	0.285	**0.847**
	This instructor explains the basic swing movement.	0.188	0.136	0.301	**0.782**
Eigenvalues variance (%)		6.711	2.476	1.264	1.009
		47.935	17.688	9.027	7.207

Analysis results exceeding the statistical threshold of 0.40 are in bold.

Additionally, reliability between the items was tested based on Cronbach’s alpha coefficients, and a Cronbach’s alpha of >0.7 was considered satisfactory: (a) expertise (basic principle), α = 0.857; (b) expertise (personality & ethics), α = 0.855; (c) expertise (performance), α = 0.941; (d) expertise (teaching skills), α = 0.929; (e) instructor credibility, α = 0.964; and (f) lesson participation intention, α = 0.958. The results showed that all the factors used in this study had satisfactory statistical reliability.

### 3.2 Multivariate analysis of variance

A factorial multivariate analysis of variance was conducted to investigate the differences among the groups (i.e., the group with an amateur teacher, the group with a semi-professional, and the group with a PGA professional) using three dependent variables (i.e., instructor expertise, instructor credibility, and lesson participation intention).

The Wilks’ Lambda multivariate *F* statistic found a statistically significant main effect of age on the dependent variables [Wilks’ *Λ* = 0.387, *F*(12, 292) = 5.837, *p* < 0.05]. Specifically, the univariate tests for performance, *F* = 16.544, *p* < 0.001, personality and ethics, *F* = 2.487, *p* > 0.05, teaching skills, *F* = 7.216, *p* < 0.01, basic principle, *F* = 0.479, *p* > 0.05, instructor credibility, *F* = 14.661, *p* < 0.001, and lesson participation intention, *F* = 12.592, *p* < 0.001 based on the independent variable were found. As mentioned previously, statistically significant differences were reported among the three groups. To verify the paired groups that were statistically significant, additional *post-hoc* analyses were performed. Detailed statistical results (MANOVA and *post-hoc* analysis) including the mean scores and standard deviations for each factor by group are listed in Tables 4–6.

**Table 4:** Results of the multivariate analysis of variance among three groups.

Variables	Sub-factors	*df*	*F*	*p*	*η^2^*	*Post-hoc*
Expertise	Performance	2	16.544	0.000^***^	0.181	a < b < c
	Personality and ethics	2	2.487	0.087	0.032	–
	Teaching skill	2	7.216	0.001^**^	0.088	a < b, c
	Basic principle	2	0.479	0.620	0.006	–
Instructor credibility		2	14.661	0.000^***^	0.164	a < b < c
Lesson participation intention		2	12.592	0.000^***^	0.144	a < b, c

^*^p < 0.05, ^**^p < 0.01, ^***^p < 0.001.

**Table 5:** Mean scores and standard deviations of dependent variables for each group.

	1	2	3	4	5	6
Group 1	3.015 (1.13)	4.163 (0.72)	3.304 (1.08)	4.000 (0.66)	3.304 (1.10)	3.010 (1.15)
Group 2	3.500 (0.99)	4.353 (0.66)	3.912 (0.93)	4.137 (0.84)	3.824 (1.02)	3.927 (0.89)
Group 3	4.083 (0.63)	4.065 (0.60)	3.882 (0.68)	4.078 (0.60)	4.314 (0.64)	3.716 (0.83)

Group 1, amateur; Group 2, semi-pro; Group 3, tour pro; 1, performance; 2, personality and ethics; 3, teaching skill; 4, basic principle; 5, instructor credibility; 6, lesson participation intention.

**Table 6:** Results of *post-hoc* analysis.

		1	2	3	4	5	6
G1	G2	0.036^*^	0.354	0.004^**^	0.622	0.023^*^	0.000^***^
	G3	0.000^***^	0.756	0.007^**^	0.856	0.000^***^	0.001^**^
G2	G1	0.036^*^	0.354	0.004^**^	0.622	0.023^*^	0.000^***^
	G3	0.009^**^	0.094	0.987	0.916	0.034^*^	0.546
G3	G1	0.000^***^	0.756	0.007^**^	0.856	0.000^***^	0.001^**^
	G2	0.009^**^	0.094	0.987	0.916	0.034^*^	0.546

^*^p < 0.05, ^**^p < 0.01, ^***^p < 0.001; G1, amateur; G2, semi-pro; G3, tour pro; 1, performance; 2, personality and ethics; 3, teaching skill; 4, basic principle; 5, instructor credibility; 6, lesson participation intention.

## 4 Discussion

This study investigated the existence of placebo and nocebo effects according to the golf instructor’s certification level, while focusing on the differences in the perception of instructor expertise, instructor credibility, and lesson participation intention among amateur golfers. The results found a partially significant difference in the perception of instructor expertise among amateur golfers, depending on the instructor’s certification level. In terms of performance, the semi-pro group rated the instructor higher than the aspiring pro group, whereas the tour pro group rated the instructor higher than the semi-pro group. In terms of teaching skills, the semi-pro and tour pro groups rated the instructor higher than the aspiring pro group, but the semi-pro and tour pro groups showed no significant differences. This study also found no significant differences in terms of personality and basic principle according to the instructor’s certification level.

These results suggest that the culture of academic elitism that values the certification level of an instructor over a career persists in Korea. In their analysis of the significant factors in selecting a golf instructor for members at a golf driving range, Hwang et al. (2008) reported that certification level (42.87%) is perceived as being far more important than experience (17.76%). Moreover, previous research (Park and Joo, 2008; Ko and Won, 2010) has shown that female golfers and high-income earners are rated higher, which are partially consistent with the findings of this study. However, Han and Seo (2020), who investigated the criteria for selecting instructors among professional Korean golfers, reported that communication skills, teaching methods, and instructor credibility are important factors. Therefore, unlike amateurs, professional golfers consider various factors when selecting instructors.

The participants of this study perceived instructors with a higher certification level as having higher levels of expertise, although instructor expertise does not necessarily mean having plenty of experience as a player (Kim, 2004). Bereiter and Scardamalia (1993) and Kuchinke (1997) argued that expertise cannot simply be defined by external status or extensive knowledge, and the process of developing expertise is poorly comprehended. Additionally, expertise can be developed through continuous effort, and it requires a combination of objective qualities, including knowledge and skills, as well as subjective qualities, such as personality, character, and temperament (Frensch and Sternberg, 1989; Ericsson and Smith, 1991; Germain and Tejeda, 2012). Therefore, to become a professional instructor, one must make constant efforts to build expertise in various aspects, rather than merely attain certification.

Perceiving an instructor’s expertise based on certification affects instructor credibility and lesson participation intention. The results showed that the level of trust in a tour pro was the highest, followed by that in a semi-pro and an amateur. This study found a small difference in lesson participation intention between the tour and semi-pro groups. Meanwhile, the difference between the two groups and the amateur group was statistically significant. Although trust is accumulated through long-term interaction with students, in this short-term study, the tour pro group showed high levels of trust and willingness to learn.

In previous studies, the participants tended to show a higher level of trust in an instructor’s teaching skills when the instructor held the Korea Professional Golfers’ Association certification (Kim, 2011; Joo, 2020), who compared the perception of domestic and international floral design certifications, verified the difference in the level of trust and preference depending on certification acquisition by identifying learners’ increased preference for and trust in international certifications compared to domestic certifications. These results indicate that perceiving expertise differently depending on certification levels affects lesson participation intention as well as instructor credibility. People trust and want to continue taking lessons from instructors they perceive as highly professional (McAllister, 1995). Furthermore, although trust is formed based on various factors apart from ability, such as sincerity and altruism (Hur, 2010), people tend to trust an instructor’s qualifications the most.

These results imply that many people prioritize certification when choosing an instructor. Certification proves, to some extent, one’s ability and is an objective criterion for judging expertise (Mayer et al., 1995). Janák (2015) stated that experience as a professional athlete or certification, rather than one’s qualities or abilities as a coach, has been the criterion for selecting instructors. People who have this belief naturally assume that “a great player makes a great coach” and that a person with a strong athletic career will inevitably have strong coaching ability, thereby leading them to trust such instructors more because they perceive them as having a higher level of expertise. Such biases result in a psychological error that undervalues or overvalues the actual abilities of an instructor. However, sports instructors are not only trainers but also educators (Szabo, 2012) and leaders who manage and guide players for their health and happiness (Janák, 2015). Therefore, the aforementioned belief system is an error of hasty generalization. This problem stems from the academic elitism prevalent in Korean society. Jeon (2017) stated that students attending good universities prove their abilities simply by attending them, and this belief creates a social hierarchy based on educational background.

Relatively, in Western societies, these problems are less prevalent, and meritocracy is a standard practice (Brauns, 2013). Meritocracy does not consider factors other than one’s abilities in their field (Son-Hing et al., 2011). For example, David Leadbetter, Hank Haney, and Mike Bender, who are considered the major coaches in the United States golf industry, did not achieve exceptional success as players, but they developed their talent early by studying their relative fields. They are recognized as capable coaches for producing many star players, developing coaching manuals and swing correction tools, and running golf academies. These coaches are recognized for their coaching skills rather than for their experience as players. Previous studies have also pointed out the problem of judging instructor expertise and selecting coaches based on their experience or certification. Many coaches selected based on these criteria are not capable instructors and lack the professional knowledge necessary to assist in the overall development of players (Côté and Gilbert, 2009; Janák, 2015).

This study verified the psychological errors involved in judging an instructor’s abilities based on the instructor’s background. Based on the results, this study suggests that academic elitism, which is the blind belief that an instructor with a superior certification background will also have good coaching ability, be avoided and that a coach’s ability be observed and evaluated from many different angles. Furthermore, although coaching activities have been conducted based on a coach’s certification thus far, future research should focus on the practical abilities that coaches must possess.

## 5 Conclusions and limitations

This study investigated whether placebo and nocebo effects exist based on the level of certification among golf instructors, focusing on differences in the perception of instructor expertise, instructor credibility, and lesson participation intention among amateur golfers. Based on the results of this study, the following conclusions were drawn: First, this study noted a difference in perceived instructor expertise among amateur golfers, depending on the instructor’s level of certification. Specifically, the results indicated a significant difference in performance and teaching skills but not in personality and emphasis on basic principles. Second, the results revealed a significant difference in perceived instructor credibility among amateur golfers, depending on the instructor’s certification level. The level of trust in the tour pro group was the highest, whereas that in the aspiring pro group was the lowest. Third, this study observed a difference in lesson participation intention among amateur golfers, depending on the instructor’s certification level, which was higher in the semi-pro and tour pro groups than in the aspiring pro group.

Through this study, where the participants received the same lesson from the same instructor, this study verified the psychological error (placebo/nocebo effects) of perceiving instructor expertise, instructor credibility, and lesson participation intention differently, depending on the instructor’s certification level. Blind faith and prejudice based on academic elitism, which is deeply rooted in Korean society, and the psychological error of undervaluing or overvaluing a person’s ability should be avoided. A culture that judges a person based on abilities should be established. Additionally, based on the data obtained from this study, follow-up studies should be conducted to develop practical instructor capabilities and create efficient teaching environments.

The limitations of this study are as follows: First, the sample size of 153 participants may not be sufficient to generalize the results of this study. In future studies, the sample size should be increased. Second, this study involved amateur golfers, and there may be differences between amateur and professional golfers. Therefore, further studies should analyze the differences between the two groups. Third, this study was conducted to verify whether placebo or nocebo effects exist in the perception of instructor expertise, instructor credibility, and lesson participation intention among amateur golfers based on the instructor’s certification background. Therefore, qualitative research on developing coaching abilities is necessary in the future.

## Data availability statement

The original contributions presented in the study are included in the article/supplementary material, further inquiries can be directed to the corresponding authors.

## Ethics statement

The studies involving humans were approved by The Institutional Review Board (IRB) of Kyung Hee University provided ethical approval in accordance with the Declaration of Helsinki. The studies were conducted in accordance with the local legislation and institutional requirements. The participants provided their written informed consent to participate in this study.

## Author contributions

HY: Conceptualization, Formal analysis, Investigation, Writing – original draft. J-HY: Conceptualization, Data curation, Investigation, Methodology, Writing – original draft. SJ: Conceptualization, Investigation, Writing – original draft. CC: Project administration, Supervision, Writing – review & editing. C-HB: Project administration, Supervision, Writing – review & editing.
